# The Performance and Carcass Characteristics of Black Baladi Kids Fed Diets with Graded Quantities of Sweet Lupin Grain (*Lupinus angustifolius*)

**DOI:** 10.3390/vetsci9010020

**Published:** 2022-01-09

**Authors:** Belal S. Obeidat

**Affiliations:** Department of Animal Production, Faculty of Agriculture, Jordan University of Science and Technology, P.O. Box 3030, Irbid 22110, Jordan; bobeidat@just.edu.jo; Tel.: +962-2-7201000 (ext. 22214); Fax: +962-2-7201078

**Keywords:** black kids, nutrient digestibility, growth performance, feed intake

## Abstract

A study was conducted to examine how lupin grains (LUPs) feeding affected nutritional intake, digestibility, growth, and carcass characteristics in kids. A total of 24 growing black kids (initial body weight (BW) = 16.4 ± 0.49 kg) were allocated to one of three diets at random. Lupin was included in the diet at a rate of 0 (control; CON), 100 (LUP100), or 200 g/kg (LUP200) of total dry matter (DM). The trial lasted for 91 days divided into 7 and 84 days to be used for adaptation and data collection, respectively. Feed intake was evaluated daily throughout the study. At the commencement and the end of the study, each kid’s body weight was measured to determine its average daily gain (ADG). On day 70, 5 kids were chosen at random from each group to investigate nutrient in vivo digestibility and N balance. At the end of the study, all of the kids were slaughtered to examine carcass features. Nutrient intakes (neutral detergent fiber, acid detergent fiber, and ether extract) were higher (*p* ≤ 0.01) in LUP-containing diets than in the CON diet. The average daily gain was greater (*p* ≤ 0.03) in diets containing lupin grains than in the CON diet. Cost of gain ($US/kg growth) was lower (*p* = 0.004) in kids fed diets containing lupin than the CON diet. Dry matter and CP digestibility rates were greater (*p* ≤ 0.03) in lupin diets. Retained N was higher (*p* = 0.04) in lupin-containing diets than in the CON diet. Cold carcass weight was higher (*p* < 0.05) for kids consuming the LUP100 diet than the CON diet. In lupin diets, carcass cut weights were higher (*p* < 0.05). Results of the current study indicate that feeding black kids diets containing lupin grains at 100 or 200 g/kg DM basis is cost effective and would increase profitability.

## 1. Introduction

Black goats are well adapted to a wide range of environmental conditions, including protein and mineral deficits, and they are extremely effective at utilizing low-quality roughages [1]. Due to these harsh environments, high costly protein sources are accounted as a major part of their diets to meet their crude protein requirements. In Jordan, goats are considered one of the main sources in providing consumers with red meat, as they account for roughly 20–25 percent of total red meat production, and many people prefer their meat over lamb meat because of the low level of fat in their carcasses, compared with lamb carcasses [2,3,4]. Generally, black goats have wide and long ears with short curved horns for females (mature body weight = 26–52 kg) and long curved horns for males (mature body weight = 40–53 kg) [4]. In recent years, the number of goats has decreased in Jordan due to the lack of good pastures, which are the main source of nutrients for the goats. As a result of the lack of good pasture, concentrated feed forms a large part of the goat’s diet. However, farmers fell into another problem, which is the high cost of these feeds [5]. At the same time, the governments greatly reduced subsidies to farmers with cheap feed, which reduced the profits of these farmers [6]. Therefore, the best solution to the problem of the scarcity and high cost of feeds is the use of alternative feeds [5,7]. The use of alternative feeds is beneficial to reduce the cost of feed and is also useful in reducing contamination of agricultural wastes [8]. Given that crude protein is one of the most expensive components in animal nutrition, substituting any part of these high-priced feeds will have a favorable influence on farmers’ income. Fortunately, when used appropriately, agro-industrial and plant by-products have a considerable impact on the profitability of sheep farming [9]. 

The main objective of using alternative feeds is to introduce a feed ingredient that reduces the cost of diets without affecting the performance and characteristics of carcasses. Sweet lupin (*Lupinus angustifolius*) is one of these options [10,11,12]. Lupin is an important legume in human nutrition. However, when they are graded, there are large quantities that are not suitable for human consumption, with broken ones and small sizes [11]. Including them in animal feed is an appropriate step to reduce the burden of expensive feed costs. According to its chemical composition, lupin is found to be a great source of protein and energy in the diet of ruminants. [10,11,13,14]. Lupin is rich in nutrients such as containing all nine necessary amino acids, as well as other minerals [12]. In two recent studies in our laboratory, we concluded that the use of sweet lupin in feeding lactating ewes [10] and growing lambs [11] had a positive effect on profitability. This is due to their cheap prices, compared with the conventional feedstuffs.

This study hypothesized that adding different levels of sweet lupin grain may improve Baladi kids’ performance and carcass characteristics. The goal of this study was to examine how feeding lupin grain affected nutritional intake, digestibility, growth performance, and carcass features in growing black Baladi kids.

## 2. Materials and Methods

### 2.1. Animals, Housing, and Diets

In total, 24 male kids (BW = 16.4 ± 0.49 kg; age = 90.1 ± 5.08 days, SD) were purchased from a local farm and transported to Jordan University of Science and Technology’s (JUST) Animal Facility, where they were weighed, ear tagged, health assessed, and treated for internal parasites. The study area is classified as semi-arid at latitude 32°30′ N and an elevation of 510 m above sea level. Upon arrival, the kids were housed in individually shaded concrete enclosures (1.5 × 0.75 m^2^) with plastic feeders and waterers. The research lasted 91 days, with the first 7 days dedicated to adaption and the next 84 days dedicated to collecting data. 

Throughout the study period, diets were mixed biweekly, and samples were taken immediately after mixing for quality control measures to ensure chemical uniformity. Samples of diets were collected each time and saved for later analysis. At the time of mixing, the concentrate part including sweet lupin (LUP) was mixed first, and wheat straw was then mixed with them. This process was carried out to assure that the concentrate part is homogenized and mixed well. In a completely randomized design, kids were randomly allocated to one of three diet groups: (1) control (CON), (2) 100 g/kg LUP (LUP100), and (3) 200 g/kg LUP (LUP200) of dietary dry matter (DM) in partial replacement of soybean meal and barley grain (Table 1). Diets were prepared according to the National Research Council [15].

On a daily basis, diets were offered for each kid once at 8:00 a.m. ad libitum at 110% of the previous day. Clean water was offered with free access all time throughout the study. Their water containers were cleaned daily or when needed. Refusals were removed, collected, and weighed daily, then stored at −20 °C until DM, and other nutrients were evaluated to calculate daily nutritional intakes. The kids were weighed at the start of the trial and at end of the study. To avoid weighing fluctuation and compute average daily weight gain (ADG) and feed conversion ratio (FCR; DM intake: weight gain), kids were weighed immediately before morning feeding.

### 2.2. In Vivo Digestibility and N Balance Trial

On day 70 of the trial, five kids were chosen at random from each group and placed in metabolic cages (1.05 × 0.80 m^2^) to test nutritional digestibility and N balance. The kids were given a five-day window to acclimate to the metabolic crates before being monitored for five days. Cages were designed to collect all excreted feces and urine. The feces were collected, weighed, and documented on a daily basis, with 10% of the excrement stored for chemical analysis. Urine was collected in plastic containers, weighed, and recorded. Then, 5% of the collected urine was stored at −20 °C to evaluate N balance. To avoid ammonia losses, each urine sampling container included 50 mL of 6N HCL. Fecal samples were composited, and oven dried at 50 °C for each kid and saved for later analysis. Dry matter and other nutrient digestibility rates were calculated based on the following equation: ((Nutrient intake − fecal nutrient output)/nutrient intake) × 100%. In addition, retained N (g/d) was calculated by subtracting N lost in feces and urine from the N intake. Then, retention N % was calculated by dividing retained N over N intake. At the end of the digestibility and N balance trial, all kids were placed back in the same pens used during the feeding period. 

Chemical analysis was performed on fecal samples, LUP grains, diet samples, and feed refusals. To measure the DM, diet samples were dried overnight in a forced air oven at 105 °C [16]. The crude protein content was determined using the Kjeldahl method (N = 6.25). An ANKOM^2000^ fiber analyzer (ANKOM Technology Corp., Fairport, NY, USA) was used to evaluate neutral detergent fiber (NDF) and acid detergent fiber (ADF) with the modification of Van Soest procedures [17]. Metabolizable energy (Mcal/kg) was calculated based on tubular values obtained from NRC [15].

### 2.3. Slaughtering Procedure

All the kids were slaughtered at JUST in the Animal Field facilities at the Research and Training Department, Faculty of Agriculture, at the end of this study to evaluate carcass characteristics. The kids were slaughtered by qualified workers at 9:00 a.m., roughly 18 h after their last meal, using a standard slaughter method [18]. Fasted live weight was measured right before slaughter. At the slaughtering time, all kids were moved from the individual pens to the slaughterhouse in the Animal Field. Each kid was slaughtered with a knife by cutting the neck down to the bone to make sure to cut the vein and artery so that the animals will die quickly. Then, hot carcass weight was measured immediately afterward. The weight of the cold carcass was measured after it had been chilled for 24 h at 4 °C. By dividing the cold carcass weight by the fasting live weight, the dressing percentage was obtained. Non-carcass components (lung and trachea, heart, liver, spleen, and kidneys) were removed and weighed immediately after slaughtering. Linear measurements—namely, tissue depth (GR), rib fat depth (J), eye muscle width (A), eye muscle depth (B), eye muscle area, fat depth, and the longissimus dorsi muscle (C), were measured on cold carcasses one day after slaughter. The carcasses were then divided into four regions (shoulder, rack, loin, and leg cuts). After cutting, the leg cut was dissected, and the longissimus dorsi muscle was removed from the loin cut, vacuum packed, and stored at −20 °C for 2 weeks until meat quality testing.

### 2.4. Statistical Methods

The Proc MIXED procedure of SAS [19] was used to analyze data. The only treatment was included in the fixed effects for all data. The random variable was kids. Whenever fixed effects proved significant (*p* ≤ 0.05), the least-square means (LS means) were used as post hoc.

## 3. Results

In comparison with the CON diet, the LUP100 and LUP200 diets reduced the cost of eating by 11 and 22%, respectively (Table 1). The chemical content of the diets was similar, with the exception of a modest rise in NDF and ADF levels in the LUP diets when compared with the CON diet.

Table 2 shows that the consumption of dry matter and crude protein (CP) was similar among the treatment diets. Other nutrient intakes (NDF, ADF, and EE) in LUP-containing meals were higher (*p* ≤ 0.01) than in the CON diet.

Initial body weight did not differ among different diets (Table 3). However, final body weight was greater (*p* ≤ 0.02) and tended to be greater (*p* = 0.09) in LUP100- and LUP200-containing diets, respectively, versus the CON diet. In addition, ADG was greater (*p* ≤ 0.03) for lambs fed on LUP-containing diets, compared with the CON diet. The feed conversion ratio was similar among diets. The cost of gain was lower (*p* = 0.004) for lambs fed LUP-containing diets versus the CON diet.

The effects of different LUP diets on nutrient digestibility in kids were studied (Table 4). Dry matter intake was comparable among treatment diets during the digestibility trial. In LUP-containing diets, DM and CP digestibility rates were higher (*p* ≤ 0.05) than in the CON diet. Diets with NDF digestibility were similar. Acid detergent fiber digestibility improved (*p* = 0.03) in the LUP200 diet than in the CON diet. No differences were observed on N intake, N loss in feces and urine, and N retention among diets. Retained N was greater (*p* = 0.04) in lupin-containing diets, compared with the CON diet.

Fasting lives weight was not different among different diets (Table 5). Hot carcass weights (kg) tended to be greater (*p* = 0.07) for kids that consumed LUP100 than kids that consumed the CON diet, whereas LUP200 was intermediate. Cold carcass weight was greater (*p* = 0.05) for kids that consumed LUP100 than kids that consumed the CON diet, while cold carcass weight tended to be greater (*p* = 0.08) for kids that consumed LUP200 than the CON diet. The dressing percentage tended to be greater (*p* = 0.07) for LUP100 and LUP200 groups, compared with the CON group. Non-carcass components did not differ among diets. Carcass cut weights (kg) were heavier (*p* < 0.05) for kids that consumed LUP100 and LUP200 diets, compared with kids that consumed the CON diet. 

On the other hand, the linear measurements of growing black Baladi kids showed no differences as provided in Table 6.

## 4. Discussion

The aim of this study was to assess the LUP supplementation effect on black Baladi kids’ performance and carcass quality. This study experimented with alternative feeds such as LUP in ruminants’ diet to obtain requirements for gains, reduce cost, and increase profitability. Table 1 summarizes the results of the experimental diets. By replacing a portion of soybean meal and barley grains with LUP at 100 and 200 g/kg DM, the diet’s cost decreased by 11 and 22%, respectively, compared with the cost of the CON diet. This is because legume seeds, such as broken and heterogeneous LUP grains, are not consumed by humans, reducing the cost of LUP diets in feeding ruminants, especially lambs. The results are in good agreement with previous studies, in which results showed a reduction of 22% in the diet cost by replacing barley grains and soybean meal partially with LUP at 200 g/kg in the diet of lactating Awassi ewes [10] or 15 and 27% when barley grains and soybean meal is replaced with LUP at 125 and 250 g/kg, respectively, in diets of growing Awassi lambs. It is possible to reduce and stabilize the fluctuations of the diets in the cost of feeding grain and other feed sources by using LUP as an alternative [11]. Using this kind of legumes is potentially high in feeding livestock due to their cheap price and high nutrient content. 

The CP and DM contents of both the CON diet and LUP-containing diets are comparable, with the exceptions of the ADF, NDF, and EE contents, which increased in lambs given LUP diet, as compared with lambs given the CON diet. This is because the ADF, NDF, and EE contents of LUP were higher than that of soybean meal and barley grains. Similar results were obtained by other research studies when LUP was included in the diets of lactating ewes [10] and lambs [11]. In summary, including the sweet lupin grains did not impact the nutrient composition of the different diets giving a great opportunity to use such products in feeding other livestock besides black kids.

The similarity in DM and CP intake data for Baladi kids in this current study demonstrates that LUP supplementation had no negative effect on this measurement. A recent study performed by Arfaoui et al. [20] reported similar results indicated that LUP supplementation had no effect on nutrient intake, which was explained by the tolerance level of the animals to lupin alkaloids. On the other hand, Tadele et al. [21] reported an enhancement in the intake of DM, CP, and NDF for the groups of lambs supplemented with LUP grains and attributed this result to the improvement in the rumen environment of those animals due to the supplementation of lupin grains to their diets. Previous studies and results obtained herein confirmed that the use of sweet lupin grains did not show any effect on intake and palatability. 

An enhancement of ADG was observed in this current study. As the LUP level increased in the kids’ diets, ADG increased, respectively. Otmani et al. [22] found no effect of adding lupin to fattening kids’ diets on their growth performance or ADG. More researchers reported that lupin addition to diets had no influence on growth performance or gain [23,24,25]. In line with this current study findings, McGregor [26] observed an increase in cashmere buck kids’ live weight when ad libitum feeding of lupin grains during the winter season was offered. Increasing gain, in the present study, might relate to the increase in nutrient digestibility since adding lupin grains to the diets improved DM, CP, and ADF digestibility rates. The positive effect of lupin supplementation on nutrient digestibility was observed by other researchers [27,28]. Ephrem et al. [23] found that the incorporation of lupine by different amounts in the diets improved lambs’ digestibility of DM and cellulose without affecting NDF and ADF digestibility rates. Shahjalal et al. [29] reported that goats supplemented with higher protein levels diets resulted in increasing digestibility of CP and EE. Moreover, increasing the level of protein in goats’ diets stimulates microbial fermentation activity and microbial protein synthesis, accordingly increasing nutrient digestibility rates as reported by Pirzado et al. [30]. 

In comparison with the CON diet, the LUP-containing diets have lower cost–benefit. This may be because the price of LUP was lower than the cost of barley grains and soybean meals during the study time. Similarly, many research studies dealt with alternative feeds from plant or agro-industrial by-products, such as sweet lupin [10,11], olive cake [31], olive cake, and *Atriplex halimus* L. [7] obtained the same results, which provided diets that were more economically feasible and beneficial to livestock production. 

Adding lupin to the diets had no effect on the N balance, although a variation in retained N amount was detected with the lupin-supplemented groups. This might be explained by the differences in the ingested concentrate quantity by kids; moreover, based on the increase in CP digestibility in LUP groups, retained N (g/d) was positively affected and increased. Arfaoui et al. [20] found that all animals in their study had positive N balances except for the group offered mixed protein supplements (lupin with soybean), which showed lower retained N and intake. 

Carcass characteristics were improved with the LUP groups. Hot, cold carcass weight, dressing percentage, and carcass cut weights were enhanced with the LUP groups, compared with the CON group. The difference in carcass traits is related to the ADG recorded, kids supplemented with LUP diets had a greater gain, compared with the CON diet. Similar to those results, Facciolongo et al. [32] reported an enhancement in carcass weight of lamb groups fed lupin when compared with other groups. Otmani et al. [22], on the other hand, found no effect of adding lupin to kids’ diets on carcass characteristics including yield, hot, and cold carcass weights. Similarly, when soybean was replaced with sweet lupin seeds in complete pellet feed for Podolian steers fed a diet containing LUP at 20%, comparable results were found in terms of carcass characteristics, as reported by Vicenty et al. [33]. Discrepancies in the impact of the LUP on carcass characteristics among different research studies could be related to the level of the inclusion, composition of the diets, and/or type of animals. 

## 5. Conclusions

In conclusion, no negative effects in performance or carcass quality were observed by the inclusion of lupin in black Baladi kids’ diets during this study. Average daily gain, cost of gain, and crude protein digestibility were improved in the sweet lupin grain diets, compared with the control diet. However, sweet lupin grains did not influence nutrient intake and digestibility, carcass characteristics, and meat quality; therefore, lupin grain can be considered as an alternative protein source during formulating rations for Baladi kids. Future research studies are needed to further ascertain that the use of sweet lupin grains in feeding other livestock is valid and beneficial in terms of economic return and productivity. 

## Figures and Tables

**Table 1:** Ingredients and chemical composition of diets fed to growing black Baladi kids.

Item	Diet ^1^		
	CON	LUP100	LUP200
Ingredients (dry matter, g/kg DM)			
Barley grain	505	460	420
Soybean meal	185	130	70
Wheat straw	290	290	290
Lupin ^2^	0	100	200
Salt	10	10	10
Limestone	9	9	9
Vitamin–mineral premix ^3^	1	1	1
Cost ($US/1000 kg) ^4^	392	349	307
Nutrients			
Dry matter, g/kg DM	902	916	920
Crude protein, g/kg DM	155	156	155
Neutral detergent fiber, g/kg DM	327	355	378
Acid detergent fiber, g/kg DM	143	161	176
Ether extract, g/kg DM	10	11	11
Metabolizable energy, Mcal/kg	2.31	2.29	2.28

^1^ Diets were (1) no lupin (CON), (2) 100 g/kg lupin (LUP100), and (3) 200 g/kg lupin (LUP200). ^2^ Contained 913, 334, 195, 156, and 150 g/kg, dry matter, crude protein, neutral detergent fiber, acid detergent fiber and ether extract, respectively. Additionally, it contained 2.67 Mcal/kg metabolizable energy. ^3^ Composition per kg contained: vitamin A, 600,000 International Unit (IU); vitamin D3, 200,000 IU; vitamin E, 75 mg; vitamin K3, 200 mg; vitamin B1, 100 mg; vitamin B5, 500 mg; lysine, 0.5%; DL-methionine, 0.15%; manganese oxide, 4000 mg; ferrous sulphate, 15,000 mg; zinc oxide, 7000 mg; magnesium oxide, 4000 mg; potassium iodide, 80 mg; sodium selenite, 150 mg; copper sulphate, 100 mg; cobalt phosphate, 50 mg; dicalcium phosphate, 10,000 mg. This mix was added at the level of 1 kg per 1000 kg. ^4^ diet cost was calculated based on the ingredient’s prices during 2021. DM: dry matter.

**Table 2:** Effects of feeding lupin (LUP) on nutrient intakes of growing black Baladi kids.

Item	Diet ^1^				
	CON	LUP100	LUP200	SE ^2^	*p* Value
Nutrient intake, g/d					
Dry matter	762	840	842	33.7	0.17
Crude protein	120	131	132	5.3	0.19
Neutral detergent fiber	263 ^a^	298 ^b^	320 ^b^	12.6	0.01
Acid detergent fiber	118 ^a^	135 ^b^	147 ^b^	6.1	0.01
Ether extract	8 ^a^	9 ^b^	9 ^b^	0.3	0.004

^1^ Diets were (1) no LUP (CON), (2) 100 g/kg LUP (LUP100), and (3) 200 g/kg LUP (LUP200). Within a row, means without common letters (^a^ and ^b^) differ (*p* ≤ 0.05). ^2^ SE = Standard error.

**Table 3:** Effects of feeding lupin (LUP) on growth performance of growing black Baladi kids.

Item	Diet ^1^				
	CON	LUP100	LUP200	SE ^2^	*p* Value
Initial weight, kg	16.8	16.4	16.0	0.49	0.22
Final weight, kg	27.8 ^a^	30.6 ^b^	29.9 ^ab^	0.63	0.02
Average daily gain, g/d	129 ^a^	170 ^b^	168 ^b^	12.97	≤0.03
Feed converstion ratio	5.99	5.25	5.28	0.35	0.26
Cost of gain	2.7 ^b^	2.2 ^a^	2.0 ^a^	0.17	0.004

^1^ Diets were (1) no LUP (CON), (2) 100 g/kg LUP (LUP100), and (3) 200 g/kg LUP (LUP200). Within a row, means without common letters (^a^ and ^b^) differ (*p* ≤ 0.05). ^2^ SE = Standard error.

**Table 4:** Effects of feeding lupin (LUP) on nutrient digestibility and N balance of growing black Baladi kids.

Item	Diet ^1^				
	CON	LUP100	LUP200	SE ^2^	*p* Value
Dry matter intake, g/d	772	848	845	56.9	0.58
Digestibility, %					
Dry matter	72.9 ^a^	79.6 ^b^	81.7 ^b^	2.10	0.05
Crude protein	67.5 ^a^	76.4 ^b^	82.0 ^b^	3.26	<0.03
Neutral detergent fiber	59.9	65.0	72.1	4.08	0.17
Acid detergent fiber	53.0 ^a^	59.4 ^ab^	66.7 ^b^	2.84	0.03
N Balance					
N intake, g/kg	21.8	22.9	21.9	0.76	0.56
N loss in feces, g/kg	5.9	5.3	4.0	0.98	0.31
N loss in urine, g/kg	5.6	3.7	4.1	0.79	0.26
N retained, g/d	9. 5 ^a^	13.9 ^b^	13.9 ^b^	1.32	<0.05
N retention, %	47.4	60.8	63.0	6.31	0.23

^1^ Diets were (1) no LUP (CON), (2) 100 g/kg LUP (LUP100), and (3) 200 g/kg LUP (LUP200). Within a row, means without common letters (^a^ and ^b^) differ (*p* ≤ 0.05). ^2^ Standard error.

**Table 5:** Effects of feeding lupin (LUP) on carcass, non-carcass components, and carcass cut weights of growing black Baladi kids.

Item	Diets ^1^				
	CON *n* = 8	LUP100 *n* = 8	LUP200 *n* = 8	SE ^2^	*p* Value
Fasting live weight (kg)	26.9	29.3	28.2	1.44	0.37
Hot carcass weight (kg)	13.0 ^a^	14.8 ^b^	13.8 ^ab^	0.67	0.07
Cold carcass weight (kg)	12.6 ^a^	14.4 ^b^	13.8 ^ab^	0.69	0.05
Dressing percentage	46.7 ^a^	49.4 ^b^	49.2 ^b^	0.87	0.08
Non-carcass components (kg) ^3^	1.3	1.4	1.5	0.07	0.17
Carcass cut weights (kg) ^4^	11.9 ^a^	13.3 ^b^	13.2 ^b^	0.69	≤0.05

^1^ Diets were (1) no LUP (CON), (2) 100 g/kg LUP (LUP100), and (3) 200 g/kg LUP (LUP200). Within a row, means without common letters (^a^ and ^b^) differ (*p* ≤ 0.05). ^2^ Standard error. ^3^ Non-carcass components (heart, liver, spleen, kidney, and lungs and trachea). ^4^ Carcass cut (shoulder, racks, loins, and legs).

**Table 6:** Effects of feeding lupin (LUP) on linear measurements of growing black Baladi kids.

Item	Diets ^1^				
	CON *n* = 8	LUP100 *n* = 8	LUP200 *n* = 8	SE ^2^	*p* Value
Leg fat depth (*L3*) (mm)	1.3	1.1	1.0	0.126	0.28
Tissue depth (*GR*) (mm)	8.1	8. 6	9.1	0.545	0.27
Rib fat depth (*J*) (mm)	1.2	1.1	1.4	0.128	0.24
Eye muscle width (*A*) (mm)	41.8	44.5	43.2	1.192	0.22
Eye muscle depth (*B*) (mm)	16.9	18.4	18.3	0.693	0.14
Fat depth (*C*) (mm)	1.0	1.0	1.0	0.0094	0.30
Shoulder fat depth (*S2*) (mm)	1.0	1.0	1.0	0.0071	0.93

^1^ Diets were (1) no LUP (CON), (2) 100 g/kg LUP (LUP100), and (3) 200 g/kg LUP (LUP200). ^2^ Standard error.

## Data Availability

The raw data have not been published or stored anywhere else; however, they are available upon request from Belal Obeidat.
